# Separate and combined associations of physical activity and obesity with lipid-related indices in non-diabetic and diabetic patients

**DOI:** 10.1186/s12944-019-0987-6

**Published:** 2019-02-12

**Authors:** Yongliang Zhang, Jian Yang, Jun Ye, Qiao Guo, Weimin Wang, Yining Sun, Qiang Zeng

**Affiliations:** 1 Institute of Health Management, Chinese PLA General Hospital, No.28, Fuxing Road, Haidian District, Beijing, 100853 People’s Republic of China; 2 Research Center for Information Technology of Sports and Health, Institute of Intelligent Machines, Chinese Academy of Sciences, Hefei, 230031 Anhui People’s Republic of China; 3 National Clinical Research Center for Geriatric Diseases, Beijing, 100853 People’s Republic of China; 4 University of Science and Technology of China, Hefei, 230026 Anhui People’s Republic of China

**Keywords:** Physical activity, Obesity, Type 2 diabetes, Dyslipidemia, Triglyceride, High-density lipoprotein cholesterol

## Abstract

**Background:**

This study evaluated the separate and combined associations of physical activity and obesity with blood lipids in Chinese adults with and without diabetes.

**Methods:**

Data of 17,535 participants aged 18 to 78 years old were collected. Physical activity was categorized as inactive (low) or active (moderate or high) according to the International Physical Activity Questionnaire. Linear and logistic regression analyses were performed to investigate the associations of physical activity and obesity with lipid-related indices.

**Results:**

Compared with physically active participants, inactive participants had higher triglyceride (TG) level, lower high-density lipoprotein cholesterol (HDL-C) level, and higher odds ratios for abnormal TG and HDL-C. Compared with non-obese participants, obese participants had higher levels of total cholesterol (TC), TG and low-density lipoprotein cholesterol (LDL-C), lower HDL-C level, and higher odds ratios for the four abnormal lipid indices. Inactive obese participants had highest levels of TC, TG and LDL-C, lowest HDL-C level, and highest odds ratios compared to the other groups. No significant associations were found between obesity and TC, LDL-C in patients with diabetes.

**Conclusions:**

Irrespective of diabetes, physical inactivity and obesity were associated with the presences of abnormal TG and HDL-C. Moreover, there were additive effects on blood lipids when physical inactivity and obesity co-occur.

## Background

Dyslipidemia contributes to the increased risk of cardiovascular morbidity and mortality [1]. In recent decades, the prevalence of dyslipidemia has increased dramatically in China [2, 3]. People with type 2 diabetes (T2D) had worse blood lipids than those without T2D [4].

Physical activity could bring benefits to the serum lipid levels to some extent [5]. Previous studies indicated that higher physical activity level would cause a decrease in triglyceride (TG) level and an increase in high-density lipoprotein cholesterol (HDL-C) level [6–8]. Physical inactivity led to an increase in total cholesterol (TC) and low-density lipoprotein cholesterol (LDL-C) [8]. Ritti-Dias’s study found that physical activity level was negatively associated with non-HDL concentrations in overweight and obese individuals [9]. Moreover, it was reported that sedentary behavior time was unfavourably associated with TC, TG and LDL-C [10, 11].

Obesity has been regarded as a risk factor for dyslipidemia, which consisted of increased TG, decreased HDL-C with HDL dysfunction and normal or slightly increased LDL-C with increased small dense LDL [12, 13]. It was indicated that body mass index (BMI) correlated positively with the levels of TC, TG and LDL-C, but negatively with HDL-C level [14]. It was also reported that increasing BMI increased risks for hypertriglyceridemia, high LDL-C and low HDL-C [15].

Most studies have examined associations of physical activity or obesity alone with lipid-related indices, including one single factor’s effect among two groups of subjects for comparison. Nevertheless, there are few studies to investigate the joint effects of physical activity and obesity on lipid-related indices [16, 17]. For example, Anderson et al. found that physical activity could attenuate some of the risk for metabolic syndrome associated with a higher BMI [17]. Furthermore, it is unclear whether the relationships of physical activity and obesity with blood lipids in diabetic patients differ from those in non-diabetic patients. Therefore, we intended to examine the individual and combined associations of physical activity and obesity with lipid-related indices in cohorts of non-diabetic and diabetic Chinese adults.

## Methods

### Participants and groups

**Fig. 1:**
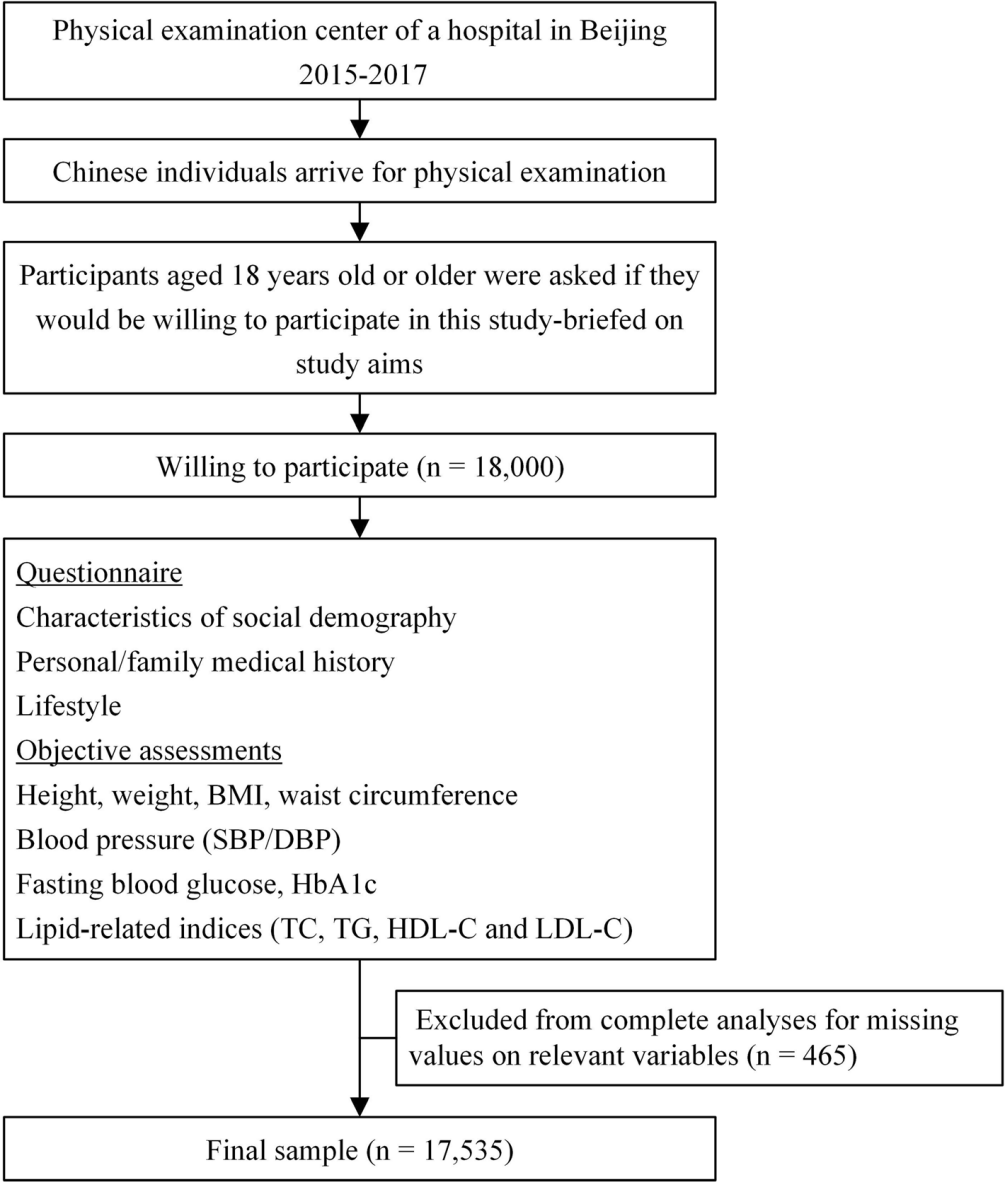
Study participants flow chart

A total of 18,000 potential participants without statins aged 18 years old or older from the physical examination center of Chinese PLA General Hospital in Beijing were screened. We excluded 465 individuals for missing values on relevant variables. Finally, 17,535 participants aged from 18 to 78 (48.6 ± 7.8) years old were included in our analyses. A flow chart of participants is shown in Fig. 1. Informed consent was obtained from all participants after detailed experimental procedures were explained. This study was approved by the Institutional Ethics Committee of Chinese PLA General Hospital and was performed in accordance with the ethical standards.

Based on estimated physical activity and the diagnosis of obesity and T2D, participants were grouped into one of the following eight groups: non-diabetic active and non-obese (*n* = 6041), non-diabetic inactive and non-obese (*n* = 6993), non-diabetic active and obese (*n* = 1159), non-diabetic inactive and obese (*n* = 1489), diabetic active and non-obese (*n* = 790), diabetic inactive and non-obese (*n* = 607), diabetic active and obese (*n* = 243), diabetic inactive and obese (*n* = 213).

### Data collection and measurements

In the physical examination center, a questionnaire was administered among participants by well-trained investigators via face-to-face interviews. The following data were collected: characteristics of social demography, medical history, and information about lifestyle including physical activity, cigarette smoking and alcohol consumption.

After the interview, participants were taken through a medical examination. Anthropometric measurements (body weight, height and waist circumference (WC)) were measured by trained nurses, according to standardized procedures [18]. BMI was then calculated as body weight (unit: kg) divided by the square of height (unit: m) using Quetlet’s index. After a period of 5-min rest in a quiet room, the systolic blood pressure (SBP) and diastolic blood pressure (DBP) were measured on the right upper arm in sitting position, using an automatic machine (AND TM-2655P, Japan). Venous blood samples were collected after an overnight fast of at least 10 h. All measurements were performed in air-conditioned room (22–26 °C) of the examination center, between 8:00 and 11:00 in the morning. Fasting plasma glucose (FPG), TC, TG, HDL-C and LDL-C levels were assayed by using a fully automated biochemistry analyzer (Roche Cobas 6000, Germany). Hemoglobin A_1c_ (HbA_1c_) was assayed with an automated glycohemoglobin analyzer (TOSOH HLC-723G8, Japan).

### Physical activity

The overall physical activity level of a person was assessed by using the International Physical Activity Questionnaire (IPAQ short form) [19]. Participants were asked to indicate the frequency (number of days) and duration (minutes) that they spent in three types of activity (walking, moderate and vigorous activities) in the past seven days. According to the protocol for IPAQ short form, we computed a metabolic equivalents of energy (MET)·minutes/week for each activity (3.3 × minutes × days for walking, 4.0 × minutes × days for moderate activity and 8.0 × minutes × days for vigorous activity). Total physical activity was the sum of walking, moderate (≥3 MET and < 6 MET) and vigorous (≥6 MET) activities in MET·minutes/week. Overall physical activity for each participant was classified as low, moderate or high level, which was defined in IPAQ short form. The participants with low level of physical activity were defined to be inactive, otherwise they were active.

### Diagnostic criteria and definition

T2D was defined as HbA_1c_ at least 6.5%, FPG at least 7.0 mmol/L and/or currently under diabetes treatment [20]. Obesity was defined as having a BMI at least 28.0 kg/m^2^ [21]. Hypertension was defined as SBP/DBP at least 140/90 mmHg and/or currently under medical treatment for hypertension [22]. Cut-off values that were often used for high TC, TG and LDL-C were 5.18 mmol/L, 1.70 mmol/L, 3.37 mmol/L, and for low HDL-C was 1.04 mmol/L, according to the clinical definition of metabolic syndrome by the National Cholesterol Education Program Adult Treatment Panel III [23]. Ischemic cardiovascular disease (ICVD) was defined as having prior physician-diagnosis of coronary heart disease, angina or myocardial infarction.

### Statistical analysis

Statistical analyses were performed with SPSS version 21.0 (SPSS Inc., IBM, USA). Continuous variables were shown as mean ± standard deviation (SD). And differences between study groups were evaluated by one-way analysis of variance (ANOVA). Categorical variables were presented as percentage and compared using Chi-square test. Stepwise multiple linear regression analysis was used to estimate the relationships between lipid-related indices (dependent variables) and BMI or WC (independent variables), adjusted for age, gender, smoking and drinking (covariates). The participants were divided into active and inactive, and regression coefficients for BMI or WC were calculated separately within each stratum. To examine whether the regression coefficients differed significantly by physical activity levels, we evaluated the interactions of physical activity with BMI and WC. In the forward binary logistic regression analysis, TC, TG, HDL-C and LDL-C were transformed into binary variables according to the abnormal cut-off values. Then, the abnormal lipid indices were set as the dependent variables separately, other variables including age, gender, smoking and drinking were set as covariate, physical activity and obesity were set as independent variables. For both the non-diabetic condition and the diabetic condition, with active non-obese group as reference, odds ratios (ORs) in the other groups were calculated. Two-sided *P*-values < 0.05 were considered to be statistically significant.

## Results

### Basic clinical characteristics

The basic characteristics of the study participants are given in detail in Table 1. Among the total participants, 75.1% (13,161/17,535) were male, 10.6% (1853/17,535) had T2D. Diabetic participants were older and had a lower percentage of women than non-diabetic participants within the same group. Among non-obese groups, the proportion of participants with smoking or drinking habits was higher in diabetic participants than that in non-diabetic participants. Diabetic participants had higher prevalence of hypertension and ICVD than non-diabetic participants within the same group.

**Table 1:** Basic socio-demographic and clinical data of the study participants (*n* = 17,535), China, 2015–2017

Group	Active and non-obese		Inactive and non-obese		Active and obese		Inactive and obese	
Condition	Non-diabetic	Diabetic	Non-diabetic	Diabetic	Non-diabetic	Diabetic	Non-diabetic	Diabetic
n	6041	790	6993	607	1159	243	1489	213
Age (years)	49.9 ± 8.0	53.0 ± 7.5^***^	47.2 ± 7.5^ǂǂǂ^	51.0 ± 6.9^***ǂǂǂ^	48.9 ± 7.9^ǂǂǂ × ××^	51.1 ± 6.9^***ǂǂǂ^	46.3 ± 7.3^ǂǂǂ × × × †††^	47.9 ± 6.4^**ǂǂǂ × × × †††^
Female (%)	30.9	14.4^***^	28.5	11.7^***^	12.5	8.6	10.5	4.7^**^
Smoking (%)	23.3	32.0^***^	31.0	38.6^***^	32.7	35.4	40.0	45.5
Drinking (%)	49.4	55.2^**^	56.4	62.3^**^	66.1	63.8	70.1	67.1
Hypertension (%)	22.7	42.3^***^	20.4	41.5^***^	43.0	63.8^***^	41.6	58.7^***^
ICVD (%)	2.6	5.7^***^	2.0	5.9^***^	3.0	7.0^**^	2.6	6.1^**^
Weight (kg)	68.97 ± 10.01	72.73 ± 9.24^***^	69.63 ± 10.32^ǂǂǂ^	73.31 ± 8.20^***^	87.91 ± 9.48^ǂǂǂ × ××^	88.16 ± 8.32^ǂǂǂ × ××^	88.90 ± 9.45^ǂǂǂ × × × †^	90.61 ± 9.84^*ǂǂǂ × × × ††^
Height (cm)	169.2 ± 7.8	170.8 ± 7.3^***^	170.0 ± 7.7^ǂǂǂ^	171.3 ± 6.7^***^	171.2 ± 7.1^ǂǂǂ × ××^	171.3 ± 6.4	171.9 ± 7.0^ǂǂǂ × × × †^	172.9 ± 6.6^ǂǂǂ × ×†^
BMI (kg/m^2^)	23.99 ± 2.35	24.86 ± 2.05^***^	24.00 ± 2.45	24.94 ± 1.94^***^	29.95 ± 2.03^ǂǂǂ × ××^	30.00 ± 1.92^ǂǂǂ × ××^	30.04 ± 1.95^ǂǂǂ × ××^	30.27 ± 2.20^ǂǂǂ × ××^
WC (cm)	85.3 ± 8.1	89.6 ± 7.0^***^	85.9 ± 8.4^ǂǂǂ^	90.0 ± 6.3^***^	99.7 ± 7.0^ǂǂǂ × ××^	101.1 ± 7.0^**ǂǂǂ × ××^	100.3 ± 7.1^ǂǂǂ × × × †^	102.2 ± 6.8^**ǂǂǂ × ××^
SBP (mmHg)	121.4 ± 14.7	125.3 ± 15.0^***^	120.1 ± 14.6^ǂǂǂ^	125.4 ± 14.8^***^	131.4 ± 14.7^ǂǂǂ × ××^	131.5 ± 14.9^ǂǂǂ × ××^	129.9 ± 13.3^ǂǂǂ × × × ††^	130.2 ± 12.7^ǂǂǂ × ××^
DBP (mmHg)	81.7 ± 10.8	83.5 ± 10.2^***^	81.2 ± 11.0^ǂ^	83.9 ± 10.3^***^	88.9 ± 10.0^ǂǂǂ × ××^	88.4 ± 10.1^ǂǂǂ × ××^	88.6 ± 10.4^ǂǂǂ × ××^	88.5 ± 9.6^ǂǂǂ × ××^
FPG (mmol/L)	5.58 ± 0.93	7.96 ± 2.17^***^	5.54 ± 0.85	8.34 ± 2.53^***ǂǂǂ^	5.85 ± 0.88^ǂǂǂ × ××^	8.39 ± 2.35^***ǂǂǂ^	5.85 ± 1.01^ǂǂǂ × ××^	8.84 ± 2.68^***ǂǂǂ × × × †††^
HbA_1c_ (mmol/L)	5.74 ± 0.55	7.15 ± 1.19^***^	5.70 ± 0.52^ǂǂǂ^	7.27 ± 1.38^***ǂǂǂ^	5.88 ± 0.59^ǂǂǂ × ××^	7.25 ± 1.25^***ǂ^	5.87 ± 0.60^ǂǂǂ × ××^	7.50 ± 1.39^***ǂǂǂ × × × †††^

Data are expressed as means (SD), or percentages

Smoking, smoked every day and had smoked for more than 6 months; Drinking, drink alcohol at least once per week and had drunk for more than 6 months

ICVD, ischemic cardiovascular disease; BMI, body mass index; WC, waist circumference; SBP, systolic blood pressure; DBP, diastolic blood pressure; FPG, fasting plasma glucose; HbA_1c_, hemoglobin A_1c_; TC, total cholesterol; TG, triglyceride; HDL-C, high-density lipoprotein cholesterol; LDL-C, low-density lipoprotein cholesterol

^*^P < 0.05, ^**^P < 0.01, ^***^P < 0.001 vs. same group, diferent condition; ^ǂ^*P* < 0.05, ^ǂǂ^*P* < 0.01, ^ǂǂǂ^*P* < 0.001 vs. active and non-obese group, same condition; ^×^*P* < 0.05, ^××^*P* < 0.01, ^× × ×^*P* < 0.001 vs. inactive and non-obese group, same condition; ^†^*P* < 0.05, ^††^*P* < 0.01, ^†††^*P* < 0.001 vs. active and obese group, same condition

### Comparison of mean levels of blood lipid-related indices between groups

**Fig. 2:**
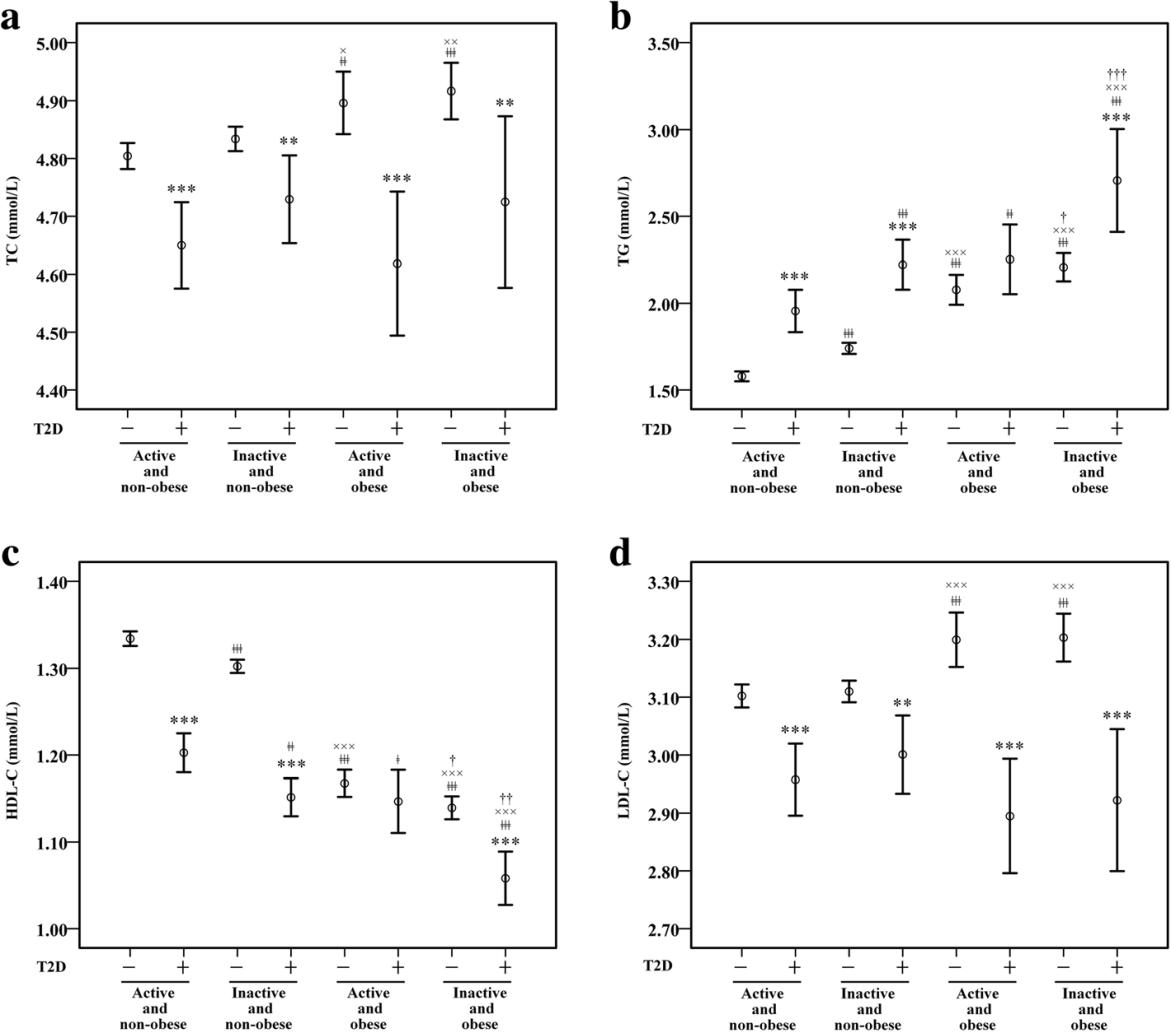
Mean levels of blood lipid-related indices. **a** TC (mmol/L), **b** TG (mmol/L), **c** HDL-C (mmol/L), **d** LDL-C (mmol/L). Error bars represented as mean ± SE. ^*^P < 0.05, ^**^P < 0.01, ^***^P < 0.001 vs. same group, different condition; ^ǂ^*P* < 0.05, ^ǂǂ^*P* < 0.01, ^ǂǂǂ^*P* < 0.001 vs. active and non-obese group, same condition; ^×^*P* < 0.05, ^××^*P* < 0.01, ^× × ×^*P* < 0.001 vs. inactive and non-obese group, same condition; ^†^*P* < 0.05, ^††^*P* < 0.01, ^†††^*P* < 0.001 vs. active and obese group, same condition

Figure 2 shows the mean levels of TC, TG, HDL-C and LDL-C. In Fig. 2a and d, obese participants had significantly higher levels of TC and LDL-C compared to non-obese participants, in non-diabetic condition, but not in diabetic condition. The levels of TC and LDL-C were lower in diabetic participants than those in non-diabetic participants within the same group (all *P* < 0.01). Diabetic participants had a higher TG level and a lower HDL-C level compared with non-diabetic participants in each group except active obese group (Fig. 2b and c). Inactive participants had significantly higher TG level and significantly lower HDL-C level compared to active participants. Obese participants had significantly higher TG level and significantly lower HDL-C level compared to non-obese participants. TG level was highest in inactive obese group, whereas HDL-C level was lowest.

### Correlations of BMI or WC with blood lipid-related indices

Table 2 presents regression slopes of TC, TG, HDL-C and LDL-C levels versus BMI and WC, stratified by physical activity. In non-diabetic participants, we observed that BMI was positively correlated with TC, TG and LDL-C, whereas negatively correlated with HDL-C. In diabetic participants, BMI was positively correlated with TG, whereas negatively correlated with HDL-C. Similar relationships were observed between WC and the four lipid indices. Table 2 also shows the significance of the interaction between physical activity level and body size on blood lipid-related indices. Higher physical activity level significantly suppressed the relationships of BMI and WC with TC, TG and LDL-C in non-diabetic participants. The reduced BMI and WC effects on TG with physical activity were also observed in diabetic participants.

**Table 2:** Regression slopes for lipid-related indices (dependent variables) vs. BMI and waist circumference (independent variables) adjusted for age, gender, smoking and drinking, stratified by physical activity

Physical activity	Dependent variable			
	TC (mmol/L)	TG (mmol/L)	HDL-C (mmol/L)	LDL-C (mmol/L)
Independent variable: BMI (kg/m^2^)				
*Non-diabetic*				
Active	0.0159 ± 0.0035^***^	0.0798 ± 0.0045^***^	− 0.0311 ± 0.0011^***^	0.0215 ± 0.0030^***^
Inactive	0.0191 ± 0.0032^***^	0.0869 ± 0.0047^***^	− 0.0304 ± 0.0010^***^	0.0232 ± 0.0027^***^
Interaction (*P*)^a^	*P* = 0.0025	*P* < 0.0001	*P* = 0.9067	*P* = 0.0294
*Diabetic*				
Active	–	0.0742 ± 0.0177^***^	− 0.0150 ± 0.0032^***^	–
Inactive	–	0.0762 ± 0.0216^***^	− 0.0158 ± 0.0029^***^	–
Interaction (*P*)		*P* = 0.0204	*P* = 0.9636	
Independent variable: WC (cm)				
*Non-diabetic*				
Active	0.0071 ± 0.0013^***^	0.0299 ± 0.0015^***^	− 0.0114 ± 0.0004^***^	0.0090 ± 0.0012^***^
Inactive	0.0088 ± 0.0012^***^	0.0328 ± 0.0018^***^	− 0.0107 ± 0.0004^***^	0.0086 ± 0.0009^***^
Interaction (*P*)	*P* = 0.0055	*P* = 0.0011	*P* = 0.5942	*P* = 0.0688
*Diabetic*				
Active	–	0.0224 ± 0.0062^***^	− 0.0050 ± 0.0012^***^	–
Inactive	–	0.0263 ± 0.0080^***^	− 0.0054 ± 0.0011^***^	–
Interaction (*P*)		*P* = 0.0233	*P* = 0.9876	

Regression coefficient ± standard error

BMI, body mass index; WC, waist circumference; TC, total cholesterol; TG, triglyceride; HDL-C, high-density lipoprotein cholesterol; LDL-C, low-density lipoprotein cholesterol

^a^The test for whether physical activity affected the relationship of lipid-related indices to BMI

^***^P < 0.001

### Associations of physical activity and obesity with abnormal TC, TG, LDL-C and HDL-C

The ORs with 95% confidence intervals (CIs) for the four abnormal lipid indices in the groups are presented in Table 3. In non-diabetic condition, obese participants had higher ORs for high TC, high TG, low HDL-C and high LDL-C compared to non-obese participants. Physically inactive participants had higher ORs for high TG and low HDL-C compared to non-obese participants. ORs for high TG and low HDL-C were highest in inactive obese group, were 2.23 (1.98–2.51) and 2.17 (1.91–2.47) respectively (*P* < 0.001 for all). In diabetic condition, physically inactive and obese participants had higher ORs for high TG and lower HDL-C which were 1.70(1.23–2.33) and 1.70(1.23–2.35) respectively (*p*<0.01 for all).

**Table 3:** Associations of physical activity and obesity with abnormal TC, TG, HDL-C and LDL-C in diabetic and non-diabetic patients

Condition Group	High TC	High TG	Low HDL-C	High LDL-C
	OR (95% CI)^a^			
*Non-diabetic*				
Active and non-obese	1.00 (reference)	1.00 (reference)	1.00 (reference)	1.00 (reference)
Inactive and non-obese	1.05 (0.98–1.13)	1.21 (1.12–1.30)^***^	1.16 (1.06–1.27)^**^	1.04 (0.97–1.12)
Active and obese	1.21 (1.06–1.38)^**^	1.79 (1.57–2.04)^***^	1.97 (1.70–2.27)^***^	1.32 (1.16–1.50)^***^
Inactive and obese	1.21 (1.07–1.37)^**^	2.23 (1.98–2.51)^***^	2.17 (1.91–2.47)^***^	1.35 (1.20–1.52)^**^
*Diabetic*				
Active and non-obese	1.00 (reference)	1.00 (reference)	1.00 (reference)	1.00 (reference)
Inactive and non-obese	1.22 (0.96–1.54)	1.35 (1.08–1.68)^**^	1.31 (1.04–1.64)^*^	1.04 (0.82–1.30)
Active and obese	0.89 (0.64–1.24)	1.66 (1.23–2.22)^***^	1.26 (1.12–1.72)^**^	0.86 (0.62–1.18)
Inactive and obese	1.13 (0.80–1.58)	1.70 (1.23–2.33)^**^	1.70 (1.23–2.35)^**^	0.96 (0.68–1.34)

OR, odds ratio; CI, confidence interval; TC, total cholesterol; TG, triglyceride; HDL-C, high-density lipoprotein cholesterol; LDL-C, low-density lipoprotein cholesterol

^a^Adjusted for age, gender, smoking and alcohol drinking

^*^P < 0.05, ^**^P < 0.01, ^***^P < 0.001

## Discussion

To our knowledge, this was the first study reporting combined associations of physical activity and obesity with blood lipids in non-diabetic and diabetic patients. The major findings from this study could be summarized as follows: (1) Physical inactivity was significantly associated with the presences of abnormal TG and HDL-C in non-diabetic and diabetic participants (Fig. 2b and c); (2) Obesity was significantly associated with the presences of the four abnormal lipid indices in non-diabetic participants (Fig. 2), while the associations disappeared in diabetic participants between obesity and the presences of abnormal TC and LDL-C (Fig. 2a and d); (3) Physical inactivity and obesity may have additive effects on TG and HDL-C in non-diabetic and diabetic participants (Table 3).

Diabetic participants had higher TG and lower HDL-C, compared to non-diabetic participants. This was consistent with the result of previous studies, which had demonstrated that the most typical lipoprotein pattern in T2D, consisted of moderate elevation in TG concentrations, and decrease in HDL-C concentrations [24]. It has been proposed that insulin deficiency or resistance, adipocytokines and hyperglycemia contributed to the risk increase of dyslipidemia, which was present even before the onset of T2D [25, 26]. While in obese group, those alterations of TG and HDL-C were not statistically significant in non-diabetic versus diabetic in our study. It may imply that obesity was more critical than diabetes for worsening TG and HDL-C levels.

In the present study, we found that obesity was positively associated with the presences of the four abnormal lipid indices, which confirmed previous reports [12, 13]. It was reported that abdominal subcutaneous fat accumulation was closely related to dyslipidemia [27]. There were significantly positive associations between physical inactivity and the presences of abnormal TG and HDL-C. The current findings were consistent with those observed in previous cross-sectional studies [6, 8] and were confirmed by prospective study [5, 28–30]. For example, Kraus et al. found that greater amount of physical activity was closely associated with improved lipids, but the intensity of exercise was less important [28]. Wang and Xu reported that physical activity promoted the reverse cholesterol transport process and lipid peroxide transport clearing, this means that physical activity not only has a positive effect on individuals with dyslipidemia, but can also help improve lipids profile [31]. Saltin and Helge pointed that physical activity increased the ability of muscles to better burn fat instead of glycogen by activation of enzymes for lipid turnover [32]. TC and LDL-C levels were not significantly different among physically active participants and inactive participants, which agreed with the result of previous studies [33, 34]. Norregaard et al. reported that six weeks of physical activity training reduced LDL-C level [30]. Pedersen Saltin pointed that high-volume/high-intensity physical activity reduced small LDL particles and increased the size of the LDL particles [35]. The relationship between physical activity and LDL-C remains unclear and complex.

It was verified that physical inactivity and obesity could bring dyslipidemia risks. The joint effects of physical inactivity and obesity had varied impact on different lipid-related indices. In obese participants, physical inactivity was related to increased TG level, decreased HDL-C level and increased ORs of high TG and low HDL-C. These findings indicated that the joint effect of physical inactivity and obesity was associated with deeper exacerbation of TG and HDL-C compared to their individual effect. It confirmed that physical inactivity could amplify some of the risk for low HDL-C associated with a higher BMI [16].

There was strong experimental evidence that physical activity could decrease the risk of developing dyslipidemia through weight loss and the current study showed that physical activity, independent of obesity, may have a beneficial effect on the lipid profile [35]. The mechanism of physical activity-induced lipid changes is still unclear, though may be partly explained by weight loss. The study described by Earnest et al. pointed that physical activity may increase blood lipid utilization hence to decrease lipids levels [36]. It was also reported that physical activity could significantly increase activity of lipoprotein lipase [37], which was responsible for chylomicrons and TG hydrolysis in granules [38]. Venkatraman et al. reported that physical activity reduce interleukin-1β, tumor Necrosis Factor-α levels in men, which may be the cause of dyslipidemia [39]. Further studies are required to decisively ascertain the mechanisms and dose-response relationship between physical activity and blood lipids.

There were some limitations that need to be considered when interpreting the findings from this study. First, the assessment of physical activity level in the present study was not based on an objective measurement by motion sensors but was derived from a physical activity questionnaire. Hence, misclassification of physical activity level may be introduced due to the recall bias. Second, although some common confounders (age, gender, smoking and drinking) had been adjusted in our analyses, the possibility still existed that unmeasured confounders might have some impact on the results. Finally, the cross-sectional study did not have a strict follow-up design. Thus, the current findings could not infer cause-and-effect relationships.

## Conclusions

The findings of this current study suggested that both physical inactivity and obesity were significantly associated with increased risks for abnormal TG and HDL-C irrespective of diabetes. Furthermore, there was an additive interaction between physical inactivity and obesity. Longitudinal studies are needed to investigate the effects and their mechanisms.

## Abbreviations

ANOVA: Analysis of variance
BMI: Body mass index
CIs: Confidence intervals
DBP: Diastolic blood pressure
FPG: Fasting plasma glucose
HbA_1c_: Hemoglobin A_1c_
HDL-C: High-density lipoprotein cholesterol
ICVD: Ischemic cardiovascular disease
IPAQ: International physical activity questionnaire
LDL-C: Low-density lipoprotein cholesterol
ORs: Odds ratios
SBP: Systolic blood pressure
T2D: Type 2 diabetes
TC: Total cholesterol
TG: Triglyceride
WC: Waist circumference
